# Exploring the psychological dimensions of poetry- and drama-based instruction in EFL learning

**DOI:** 10.3389/fpsyg.2026.1802084

**Published:** 2026-06-18

**Authors:** Mohammed Samer Barbour, Emre Debreli

**Affiliations:** Faculty of Education, Cyprus International University, Nicosia, Cyprus

**Keywords:** foreign language enjoyment, language learning, learner confidence and voice, poetry and drama in EFL, qualitative psychology in SLA

## Abstract

Recent research in second language acquisition has increasingly emphasized the psychological dimensions of language learning, particularly emotion, confidence, engagement, and learner identity. Within this context, poetry and drama-based instruction has been identified as a potentially meaningful pedagogical approach. However, adolescent learners’ psychological experiences of these practices remain underexplored, particularly in exam-oriented secondary school settings where creative language activities are often marginalized. Addressing this gap, the present qualitative study explored how adolescent learners and teachers experienced the psychological dimensions of poetry and drama-based instruction in English as a Foreign Language (EFL) classrooms. The study involved 24 final-year secondary school students aged 16–18 and six EFL teachers from two secondary schools. Data were collected through Arabic-language focus group interviews with students and semi-structured individual interviews with teachers. The data were analyzed using Braun and Clarke’s reflexive thematic analysis, informed by perspectives from positive psychology in second language acquisition and sociocultural theory. The findings revealed three interrelated themes: (1) poetry and drama as emotionally supportive practices that reduced anxiety and fostered enjoyment, (2) enhanced speaking confidence and opportunities for self-expression and learner voice, and (3) meaningful engagement shaped by both individual learner differences and institutional constraints associated with exam-oriented schooling. The findings suggest that poetry and drama can function as psychologically meaningful pedagogical spaces in which adolescent learners experience English not only as an academic subject, but also as a medium for expression, interaction, and identity exploration. At the same time, the study highlights how the psychological benefits of creative language practices remain shaped by broader institutional pressures and classroom realities. The study contributes to research on positive psychology in SLA by offering a qualitative account of how emotionally supportive and expressive classroom practices are experienced within a high-stakes EFL context. Pedagogically, the findings underscore the potential value of integrating creative practices into secondary EFL classrooms in ways that support learners’ emotional engagement, confidence, and sense of voice.

## 1 Introduction

In recent decades, research in second and foreign language learning has increasingly emphasized the importance of learners’ psychological experiences, including emotion, self-beliefs, engagement, and identity, in shaping language learning processes (Dörnyei and Ryan, 2015; MacIntyre et al., 2019). From this perspective, language learning is no longer understood solely as a cognitive endeavor focused on grammar and vocabulary acquisition, but as a deeply affective and social experience, particularly during adolescence, a developmental period characterized by heightened self-awareness, sensitivity to peer evaluation, and identity formation (Holme, 2012; Steinberg, 2014). Within this broader psychological turn in applied linguistics, creative and arts-based pedagogical approaches have gained renewed attention for their potential to support learners’ emotional and motivational engagement with language learning (Burke and Field, 2023; Jacobs, 2023).

Poetry and drama-based instruction has long been recognized as a valuable pedagogical resource in English as a Foreign Language (EFL) education (Zhang, 2024). Previous work suggests that poetry and drama can promote linguistic awareness, communicative competence, interpretive skills, and opportunities for authentic language use by engaging learners in expressive and interactive communication (Beaumont, 2022; Lazar, 1993; Paran, 2008). Drama-oriented approaches, in particular, encourage embodied learning, collaborative meaning-making, and contextualized language practice, all of which may foster greater involvement in classroom interaction (Even, 2011; Maley and Duff, 2005; McAvoy and O'Connor, 2022). Similarly, poetry has been shown to create opportunities for emotional expression, aesthetic engagement, and personal response, enabling learners to connect language learning with lived experience and subjective meaning (Hanauer, 2012). As a result, poetry and drama have increasingly been discussed not only as instructional tools, but also as practices that may shape learners’ emotional and psychological relationship with the foreign language.

Despite these potential benefits, creative language practices often occupy a marginal position in upper secondary EFL classrooms, particularly in educational systems shaped by high-stakes examinations and performance-oriented curricula. In many exam-driven contexts, including the United Arab Emirates (UAE), instructional priorities are frequently directed toward measurable linguistic outcomes, textbook completion, and preparation for standardized assessments. As a consequence, classroom activities that emphasize creativity, emotional expression, or exploratory language use may be viewed as secondary to examination preparation. For adolescent learners who are already navigating academic pressure and heightened sensitivity to evaluation, such environments can contribute to anxiety, reduced willingness to communicate, and a perception of English as primarily an academic requirement rather than a medium for interaction or self-expression (Zhang, 2024). Within these contexts, understanding how learners psychologically experience alternative and creative pedagogical practices becomes particularly important.

At the same time, research in the psychology of language learning has highlighted the central role of positive emotional experiences, such as enjoyment, confidence, and a sense of personal voice, in sustaining learner engagement and willingness to use the foreign language (Dewaele and MacIntyre, 2014; MacIntyre and Gregersen, 2012). Studies on foreign language enjoyment suggest that classroom environments characterized by supportive interaction, creativity, and learner autonomy can contribute to more meaningful and emotionally positive language learning experiences (Dewaele et al., 2019). From this perspective, poetry and drama may hold particular psychological relevance for adolescent learners because they create opportunities for expression, interaction, and identity exploration during a developmental stage in which learners are actively negotiating self-concept and social belonging. However, while previous studies have explored poetry and drama in relation to linguistic outcomes, classroom participation, or instructional effectiveness, considerably less attention has been paid to how adolescent learners themselves psychologically experience these practices. Much of the available literature relies on quantitative approaches or teacher-reported perceptions, providing limited insight into students’ subjective emotions, confidence-related experiences, and sense of self as language users. Furthermore, qualitative studies that foreground adolescent learners’ voices remain relatively scarce, particularly in exam-oriented EFL settings where creative pedagogies may be shaped by institutional constraints. Consequently, there remains a need for qualitative research that examines how poetry and drama-based instruction is experienced emotionally, socially, and psychologically by adolescent learners and teachers.

The present study addresses this gap by exploring poetry and drama-based instruction as a psychologically meaningful classroom practice situated within the realities of exam-oriented secondary education. Drawing on perspectives from positive psychology in second language acquisition and sociocultural theory, the study seeks to contribute to existing research by examining how emotional safety, confidence, learner voice, and engagement are experienced and negotiated through creative language practices. In doing so, the study extends current discussions of foreign language enjoyment and expressive pedagogy by illustrating how the psychological benefits of creative practices are shaped not only by classroom interaction, but also by broader institutional expectations and educational pressures. Practically, the study aims to provide insight for EFL teachers and curriculum designers seeking to integrate emotionally supportive and engaging language practices into secondary school contexts.

Addressing these aims, the present study adopts a qualitative interpretive approach to explore the psychological dimensions of poetry and drama-based instruction in adolescent EFL learning. Focusing on final-year secondary school students aged 16–18 and their teachers in two UAE secondary schools, the study seeks to illuminate how creative language practices are perceived, experienced, and interpreted within the broader psychological landscape of late-adolescent language learning. Guided by this aim, the study addresses the following research questions:

1. How do adolescent EFL learners describe their psychological experiences of poetry and drama-based instruction in the classroom?
2. In what ways do poetry and drama activities influence learners’ emotions, speaking confidence, engagement, and sense of self-expression in English learning?
3. How do students and teachers perceive the psychological value and challenges of poetry and drama-based instruction in exam-oriented EFL classrooms?

## 2 Literature review

### 2.1 Psychological perspectives on language learning in adolescence

Contemporary research in second and foreign language acquisition has increasingly moved beyond purely cognitive explanations of language learning to emphasize the psychological, emotional, and social dimensions of the learning experience. From this perspective, language learning is understood as a complex process shaped not only by linguistic input and instructional methods, but also by learners’ emotions, self-beliefs, social relationships, and engagement within specific educational contexts (Dörnyei and Ryan, 2015; MacIntyre et al., 2019). This shift has been particularly influential in highlighting how classroom practices can either support or hinder learners’ psychological involvement in language learning. Within this broader psychological turn, emotions have emerged as a central component of language learning. Research in positive psychology in SLA suggests that positive emotional experiences, such as enjoyment, interest, curiosity, and a sense of personal meaning, play a facilitative role in sustaining learner engagement and encouraging active language use (Dewaele and MacIntyre, 2014; MacIntyre and Gregersen, 2012). Foreign language enjoyment, in particular, has increasingly been conceptualized not simply as pleasure or entertainment, but as a complex emotional experience shaped through supportive classroom relationships, meaningful participation, and learners’ perceptions of competence and agency (Dewaele et al., 2018; Li et al., 2018). Rather than viewing emotion merely as an individual trait, recent work conceptualizes it as dynamically co-constructed through classroom interaction, task design, peer relationships, and teacher practices (Mercer and Dörnyei, 2020). As a result, pedagogical approaches that invite creativity, expression, and personal involvement are increasingly understood as psychologically significant in shaping learners’ experiences of the foreign language.

Adolescence represents a particularly important period for examining the psychological dimensions of language learning. Learners during this stage are often navigating heightened self-consciousness, concerns about peer evaluation, academic pressure, and ongoing identity development, especially in exam-oriented educational systems (Steinberg, 2014). These developmental characteristics can intensify foreign language anxiety and reluctance to participate orally, making emotional safety and confidence particularly important for classroom engagement. At the same time, adolescence is associated with increased interest in self-expression and social positioning, meaning that opportunities to communicate personal experiences and viewpoints may carry particular psychological significance for learners (Ushioda, 2011). Consequently, instructional practices that encourage experimentation with language, creativity, and personal voice may hold unique relevance for adolescent language learners.

Self-efficacy and speaking confidence have also been identified as important psychological constructs influencing learners’ willingness to communicate and participate in classroom interaction (Fan, 2022; Wang et al., 2023). Drawing on Bandura (1997) theory of self-efficacy, research in SLA suggests that learners’ beliefs about their communicative capabilities can shape participation more strongly than objective proficiency alone. Learners who perceive themselves as capable of communicating meaningfully are more likely to engage actively and tolerate linguistic uncertainty. Classroom environments that reduce fear of negative evaluation and create supportive interactional conditions are therefore more likely to foster willingness to speak and sustained engagement (Gregersen and MacIntyre, 2014; Jacobs, 2023). Importantly, confidence in foreign language learning is increasingly viewed as socially mediated rather than individually possessed. Learners’ confidence develops through interactional experiences, peer responses, classroom norms, and opportunities to participate meaningfully in communication (Mercer and Dörnyei, 2020).

These psychological perspectives suggest that language learning, particularly during adolescence, is deeply intertwined with emotion, confidence, engagement, and identity-related processes. Understanding how specific classroom practices are psychologically experienced by learners is therefore essential for developing a more nuanced understanding of language learning in secondary education contexts (Jacobs, 2023; Zhang, 2024). This perspective provides an important foundation for examining creative pedagogical approaches such as poetry and drama not merely as instructional techniques, but as practices that may shape learners’ emotional engagement, sense of self, and relationship with the foreign language.

### 2.2 Poetry and drama as psychologically meaningful practices in EFL learning

Within communicative and learner-centered approaches to language education, poetry and drama have increasingly been conceptualized as practices that engage learners not only linguistically, but also emotionally, socially, and imaginatively. Unlike highly form-focused instructional activities, poetry and drama invite learners to experience language as expressive, embodied, and personally meaningful, positioning learners as active meaning-makers rather than passive recipients of linguistic input (Hanauer, 2012; Maley and Duff, 2005). From a psychological perspective, such practices may hold particular potential for fostering emotional engagement, confidence, and meaningful participation in foreign language use (Burke and Field, 2023).

Drama-based instruction has been widely discussed as a pedagogical approach that promotes interaction, collaboration, and experiential learning. By engaging learners in role-play, performance, improvisation, and imagined contexts, drama creates opportunities for reduced self-consciousness and lowered fear of negative evaluation, as learners communicate through fictional roles rather than directly as themselves (Even, 2011; Jacobs, 2023). This distancing effect has been argued to support learners’ willingness to communicate, particularly in contexts where speaking anxiety and fear of error are prevalent (Zhang, 2024). Importantly, drama may create temporary interactional spaces in which learners feel permitted to take linguistic risks without the same level of personal exposure associated with conventional classroom participation (Jacobs, 2023). Such experiences may contribute to emotional safety and greater communicative experimentation among adolescent learners. In addition, the embodied and collaborative nature of drama activities aligns closely with sociocultural perspectives on learning, which emphasize interaction, mediation, and shared meaning-making as central to development (Vygotsky, 1978).

Poetry, similarly, has been described as a pedagogical space in which emotional resonance, aesthetic engagement, and personal response are central. Hanauer (2012) argues that engaging with poetry in the language classroom enables learners to connect linguistic forms with personal meaning, thereby fostering emotional investment and a sense of ownership over the foreign language. Through metaphor, rhythm, imagery, and interpretation, poetry may allow learners to express subjective experiences that are difficult to articulate through more transactional language activities. For adolescent learners in particular, opportunities for creative expression may support identity exploration and the development of personal voice in the foreign language (Ushioda, 2011). Rather than focusing exclusively on accuracy and performance, poetry-based activities may shift learners’ attention toward meaning, feeling, and interpretation, potentially reshaping how learners emotionally relate to English language use (Jacobs, 2023).

While previous research has highlighted various pedagogical benefits of poetry and drama, including communicative practice, cultural exposure, and increased classroom interaction (Lazar, 1993; Paran, 2008), fewer studies have explicitly examined these practices through a psychological lens. Existing work often focuses on instructional effectiveness or language skill development, offering comparatively limited insight into how learners emotionally experience these activities or how such experiences shape confidence, engagement, and identity as language users. Moreover, challenges associated with poetry and drama, such as learner resistance, performance-related discomfort, or institutional constraints, are often discussed in technical or curricular terms rather than as psychologically meaningful experiences that may influence learners’ willingness to participate (Paran, 2008).

From a positive psychology perspective, poetry and drama can be understood as pedagogical contexts that may elicit enjoyment, curiosity, meaningful participation, and emotional connection in language learning (Dewaele et al., 2019; Wu and Kabilan, 2025). However, the extent to which these psychological dimensions emerge depends not only on the activities themselves, but also on how learners interpret and negotiate their experiences within particular classroom and institutional contexts. This is particularly important in exam-oriented educational environments, where creative and expressive activities may simultaneously provide emotional support while also existing in tension with assessment-driven expectations and classroom norms. Consequently, poetry and drama-based instruction can be more productively understood not simply as instructional techniques, but as socially and psychologically situated classroom experiences.

### 2.3 Research gaps and the need for a qualitative psychological perspective

Despite growing interest in creative and arts-based approaches to EFL instruction, research examining poetry and drama has largely been dominated by quantitative designs, outcome-oriented evaluations, or teacher-centered perspectives. Many studies focus on measurable gains in language skills, participation, or attitudes, often relying on questionnaires and survey instruments that provide limited access to learners’ subjective meanings and emotional experiences. As a result, relatively little is known about how adolescent learners themselves make sense of poetry and drama-based instruction within the psychological realities of secondary education. In addition, qualitative studies that foreground learners’ voices remain relatively scarce, particularly in exam-oriented EFL settings where creative practices are frequently constrained by curricular and institutional pressures. Adolescents’ emotional responses, confidence-related experiences, identity negotiations, and perceptions of classroom safety in relation to poetry and drama have received limited empirical attention, despite their relevance to both positive psychology in SLA and adolescent developmental psychology. Teacher perspectives, while occasionally included in previous studies, are rarely examined alongside student accounts in ways that illuminate how creative pedagogies are negotiated within real classroom conditions.

Another limitation in the existing literature is the tendency to discuss positive emotional experiences in relatively decontextualized ways. While concepts such as enjoyment, confidence, and willingness to communicate are widely emphasized in positive psychology research, fewer studies have examined how these experiences are shaped by institutional expectations, examination pressures, and classroom power relations. In exam-oriented educational settings, psychologically supportive practices may coexist with structural constraints that limit learners’ participation, emotional comfort, or willingness to engage creatively. Understanding these tensions requires qualitative inquiry capable of capturing emotional nuance, ambivalence, and contextual complexity.

To address these gaps, the present study adopts a qualitative interpretive approach to explore the psychological dimensions of poetry and drama-based instruction in adolescent EFL learning. By drawing on Arabic-language focus group interviews with final-year secondary school students and semi-structured interviews with EFL teachers, the study seeks to examine how poetry and drama are experienced, interpreted, and negotiated as psychologically meaningful classroom practices within an exam-oriented educational context. Grounded in perspectives from positive psychology in SLA and informed by sociocultural views of learning, the study aims to contribute a more contextually situated understanding of emotional engagement, confidence, learner voice, and institutional constraint in adolescent EFL learning.

### 2.4 Theoretical lens

The present study is theoretically informed by two complementary perspectives: positive psychology in second language acquisition and sociocultural theory. Together, these perspectives provide a framework for understanding language learning as both an emotional and socially situated process.

Positive psychology in SLA emphasizes the role of positive emotional experiences, self-beliefs, learner strengths, and meaningful engagement in shaping language learning processes (Dörnyei and Ryan, 2015; MacIntyre et al., 2019). Rather than focusing exclusively on anxiety or learner deficits, this perspective examines how classroom experiences such as enjoyment, confidence, agency, and supportive interaction contribute to language learning and participation. Foreign language enjoyment has been identified as particularly significant because it reflects not only pleasure, but also learners’ perceptions of challenge, accomplishment, social support, and meaningful engagement within the classroom (Dewaele and MacIntyre, 2014). In the context of the present study, positive psychology provides a lens for examining how poetry and drama may create emotionally supportive and expressive learning experiences for adolescent EFL learners.

At the same time, sociocultural theory conceptualizes learning as socially mediated and constructed through interaction, participation, and collaborative meaning-making (Vygotsky, 1978). From this perspective, classroom activities are not simply vehicles for transmitting linguistic knowledge, but social spaces in which learners negotiate identity, participation, and relationships with others. Poetry and drama align closely with sociocultural views of learning because they involve interaction, imagination, role-taking, and collective engagement. These practices may therefore shape not only language use, but also learners’ emotional positioning and sense of self within the classroom.

Importantly, the integration of these perspectives allows the study to move beyond viewing emotions such as enjoyment or confidence as purely internal psychological states. Instead, emotions are understood as emerging through participation in socially and institutionally situated classroom practices. This combined framework is particularly useful for examining how emotionally supportive experiences associated with poetry and drama are shaped by broader contextual realities, including peer interaction, classroom norms, and exam-oriented educational pressures.

## 3 Method

### 3.1 Research design

The present study adopted a qualitative interpretive research design to explore the psychological dimensions of poetry and drama-based instruction in adolescent EFL learning. Qualitative methodology was considered appropriate given the study’s focus on learners’ and teachers’ subjective experiences, perceptions, and meaning-making processes, rather than on measuring predefined variables or establishing causal relationships (Creswell and Poth, 2016). In line with perspectives from positive psychology in second language acquisition, the study sought to understand how classroom practices involving poetry and drama were experienced emotionally and psychologically by adolescent learners, particularly in relation to enjoyment, confidence, engagement, and self-expression. Qualitative inquiry is especially suited to examining complex psychological phenomena that are context-dependent, socially situated, and shaped through interaction (Denzin and Lincoln, 2011).

Rather than aiming for statistical generalization, the study sought to develop a contextually grounded and interpretive understanding of how poetry and drama functioned as psychologically meaningful classroom practices within an exam-oriented secondary education context. The interpretive orientation of the study assumes that participants’ experiences and accounts are shaped by their social, cultural, and institutional environments, and that meanings are co-constructed through interaction between participants and researchers.

To capture multiple perspectives and deepen contextual understanding, the study employed a multi-perspectival qualitative design incorporating both student and teacher accounts. Student experiences were explored through focus group interviews, which enabled collective reflection and interaction among peers, while teacher perspectives were examined through individual semi-structured interviews. Rather than seeking triangulation in a positivist sense, the inclusion of multiple participant perspectives was intended to support a richer and more nuanced understanding of the phenomenon through the exploration of converging, complementary, and occasionally contrasting experiences.

### 3.2 Participants and context

The study was conducted in two secondary schools in the United Arab Emirates (UAE), where English is taught as a foreign language and educational practices are strongly shaped by standardized curricula and high-stakes assessment requirements. This context was considered particularly relevant given the academic pressures commonly experienced by final-year secondary school students and the relatively limited instructional space often allocated to creative language practices such as poetry and drama. The multilingual and multicultural nature of the UAE educational environment further highlights the importance of classroom practices in shaping learners’ emotional engagement, confidence, and willingness to use English during late adolescence.

Student participants consisted of 24 final-year secondary school students aged 16–18 who had prior exposure to poetry and/or drama-based activities in their EFL classes. A purposive sampling strategy was employed to recruit students who could reflect meaningfully on their experiences with these instructional practices (Creswell and Poth, 2016). Students and teachers were recruited from the same schools in order to provide contextual continuity between learner and teacher perspectives. The student participants represented linguistically and culturally diverse backgrounds commonly found within UAE secondary education, although Arabic was the primary shared language among participants. The sample included students with varying levels of self-reported confidence in English, allowing for a range of perspectives on poetry- and drama-based learning experiences.

The 24 students were organized into four focus groups, each consisting of six participants. This group size was selected to encourage interaction and shared reflection while ensuring that all participants had opportunities to contribute meaningfully to the discussion. Participation was voluntary, and students were informed that their participation would not affect their academic standing. Given that all student participants were minors, parental consent and student assent were obtained prior to data collection. In addition to students, six EFL teachers participated in the study. Teachers were recruited using purposive sampling based on their experience integrating poetry and/or drama activities into secondary-level EFL instruction. All participating teachers had a minimum of 5 years of teaching experience and were familiar with the curricular expectations and examination pressures associated with upper secondary education in the UAE. Teacher perspectives were included to provide contextual insight into classroom practices, institutional constraints, and observed learner responses related to poetry and drama-based instruction. Table 1 presents participant demographics.

**Table 1:** Participant demographics.

Participant group	Number of participants	Age range/experience	Contextual characteristics
Final-year secondary school students	24	16–18 years	Students from two UAE secondary schools with prior exposure to poetry and drama-based EFL activities; diverse self-reported confidence levels in English
EFL teachers	6	5–11 years teaching experience	Teachers experienced in integrating poetry and/or drama activities within exam-oriented secondary EFL classrooms

Student participants were assigned pseudonyms using the codes ST1-ST24, while teacher participants were identified using the codes T1-T6. Ages are reported alongside student excerpts where relevant to interpretation.

### 3.3 Data collection

Data were collected through student focus group interviews and teacher semi-structured interviews in order to capture multiple perspectives on the psychological dimensions of poetry and drama-based instruction in adolescent EFL classrooms. Participants’ prior exposure to poetry- and drama-based instruction included role-play activities, poetry discussions, dramatic reading, short classroom performances, and collaborative interpretation tasks incorporated into regular EFL lessons. This combination of methods allowed for an in-depth exploration of learners’ and teachers’ experiences while also enabling a richer and more nuanced understanding of the phenomenon through multiple participant perspectives.

During the study, the first author conducted the data collection as an outsider researcher and had no prior instructional or evaluative relationship with the participating schools or students. Access to the research sites was obtained through formal communication with school administrators following institutional ethical approval procedures. Particular attention was given to minimizing power imbalances during data collection, especially during student focus groups, by emphasizing the voluntary nature of participation, ensuring confidentiality, and adopting a non-evaluative conversational approach throughout the interviews.

#### 3.3.1 Student focus group interviews

Student data were gathered through semi-structured focus group interviews, which were selected to facilitate shared reflection and interaction among adolescent learners. Focus groups are particularly suitable for exploring emotional and psychological experiences because participants are able to respond to, elaborate on, and negotiate one another’s perspectives during discussion. This method also aligns with sociocultural perspectives on learning, which emphasize the co-construction of meaning through social interaction.

A total of four focus group sessions were conducted, each lasting approximately 40–60 min. The focus groups were guided by a semi-structured interview protocol designed to elicit students’ reflections on their experiences with poetry- and drama-based activities in EFL classes. Key areas of inquiry included emotional responses to these activities, perceived changes in speaking confidence and willingness to participate, experiences of engagement and self-expression, and feelings of discomfort or resistance associated with creative classroom practices.

The focus group interviews were conducted in Arabic in order to ensure that participants could express themselves comfortably and in depth. The interviews were audio-recorded, transcribed in Arabic, and subsequently translated into English by one of the researchers, who is a native speaker of Arabic and fluent in English. To enhance translation accuracy and preserve interpretive meaning, the translated transcripts and selected participant excerpts were independently cross-checked by an experienced bilingual translator familiar with educational research contexts. Particular attention was paid to preserving the emotional tone and contextual meaning of participants’ accounts during the translation process.

#### 3.3.2 Teacher semi-structured interviews

To complement student perspectives, individual semi-structured interviews were conducted with EFL teachers. Teacher interviews aimed to explore instructional intentions, observed student responses, and perceived psychological benefits and challenges associated with creative language practices. Each teacher interview lasted approximately 30–45 min and followed a flexible interview guide that allowed participants to elaborate on issues they considered particularly salient. Interview questions focused on teachers’ rationales for using poetry and drama, their observations of students’ emotional engagement and confidence, perceived constraints related to curriculum and assessment, and reflections on the feasibility of integrating creative practices in exam-oriented contexts. All teacher interviews were conducted in English, and pseudonyms were assigned to both student and teacher participants to ensure confidentiality.

### 3.4 Data analysis

The interview and focus group data were analyzed using Braun and Clarke (2006, 2021) reflexive thematic analysis. Reflexive thematic analysis was considered appropriate because of its flexibility and its suitability for exploring participants’ subjective experiences, meanings, and emotional interpretations within an interpretive qualitative framework. Consistent with Braun and Clarke’s approach, themes were understood as interpretive patterns constructed through the researchers’ active engagement with the data rather than as objective categories passively emerging from it.

Analysis began with data familiarization, during which the researchers repeatedly read the interview transcripts while recording preliminary reflections related to emotional experience, confidence, engagement, self-expression, and institutional constraint. In the second phase, initial codes were generated inductively across the dataset, focusing on segments of data that captured psychologically meaningful experiences and interpretive significance rather than frequency alone. Coding remained recursive and flexible throughout the analytic process, with codes being revised, combined, or reconsidered as interpretation developed. In the third phase, codes were examined and clustered into broader patterns of shared meaning that informed the development of candidate themes. These themes were subsequently reviewed, refined, and defined in relation to the research questions and theoretical framework of the study. Attention was given not only to common experiences across participants, but also to moments of tension, discomfort, ambivalence, and divergence within the dataset. Such accounts were retained in the analysis to preserve complexity and avoid overly simplified interpretations of poetry- and drama-based instruction.

While coding was primarily inductive, interpretation of the themes was informed by perspectives from positive psychology in SLA and sociocultural theory. In particular, the analysis considered how emotional experiences such as enjoyment and confidence were socially shaped through classroom participation, interaction, and institutional context. This theoretically informed interpretation enabled the researchers to examine poetry and drama not simply as instructional techniques, but as psychologically and socially situated classroom practices.

Consistent with reflexive thematic analysis, researcher subjectivity was treated as an analytic resource rather than a source of bias to be eliminated (Braun and Clarke, 2021). The researchers engaged in ongoing reflexive discussion throughout the analytic process, critically considering how their assumptions, theoretical orientations, and educational backgrounds may have shaped interpretation of the data. Rather than seeking coding consensus or inter-rater reliability, the analysis emphasized interpretive depth, reflexive engagement, and transparency of analytic decision-making. Analytic memos and theme development notes were maintained throughout the process to support reflexivity and methodological transparency.

### 3.5 Trustworthiness and ethical considerations

To enhance the trustworthiness of the study, the research followed established qualitative principles related to credibility, dependability, reflexivity, and transparency (Lincoln and Guba, 1985). Credibility was supported through the inclusion of multiple participant perspectives, including both student and teacher accounts, which enabled a richer understanding of the psychological dimensions of poetry and drama-based instruction across different classroom roles and experiences. The inclusion of illustrative excerpts throughout the findings further supports transparency by grounding interpretations in participants’ accounts.

Dependability was addressed through the maintenance of a systematic and clearly documented analytic process. Data collection procedures, coding decisions, theme development, and reflexive observations were documented throughout the research process to provide a transparent audit trail. Reflexivity was also central to the study, with the researchers continuously examining how their backgrounds, assumptions, and theoretical commitments may have shaped interpretation of the data.

Given the cross-language nature of the study, additional attention was paid to linguistic and interpretive trustworthiness during the translation process. The use of bilingual transcription and independent translation cross-checking was intended to preserve participants’ intended meanings as closely as possible while acknowledging that some emotional nuance may inevitably shift across languages.

Ethical considerations were carefully addressed throughout the study. Ethical approval was obtained from the Cyprus International University Ethics Committee, and all procedures adhered to relevant ethical guidelines for research involving human participants. Participation was voluntary, and informed consent was obtained from all participants. As student participants were minors, parental consent and student assent were secured prior to data collection. Participants were informed of their right to withdraw from the study at any stage without consequence.

To protect confidentiality and anonymity, pseudonyms were assigned to all participants, and identifying information was removed from transcripts and reports. Particular care was taken to create a respectful and non-evaluative interview environment in which students felt comfortable expressing both positive and negative experiences openly.

## 4 Findings

This section presents the findings of the qualitative analysis of student focus group interviews and teacher semi-structured interviews. Drawing on reflexive thematic analysis, three interconnected themes were identified that capture how adolescent learners and teachers experienced the psychological dimensions of poetry- and drama-based instruction in EFL classrooms. The themes reflect students’ emotional experiences, perceptions of confidence and voice, and patterns of engagement shaped by both opportunities and constraints.

### 4.1 Emotional safety and enjoyment in poetry and drama-based learning

#### 4.1.1 Reduced fear of mistakes and evaluation

Across the focus group discussions, students consistently described poetry and drama activities as emotionally different from conventional English lessons. Many participants associated these activities with reduced anxiety, lower fear of mistakes, and a greater sense of emotional comfort during classroom participation. In contrast, regular exam-oriented lessons were frequently described as stressful, highly evaluative, and strongly focused on correctness.

Several students explained that drama activities helped them feel less exposed when speaking English because attention was directed toward the activity itself rather than linguistic accuracy. One student reflected:

*“In normal lessons, I think too much before speaking because I am afraid the teacher or other students will notice my mistakes. But during drama, everybody is focused on the scene or the character, so I feel less pressure. Even if my English is not perfect, I still continue speaking.”* (ST3, age 17)

Another participant described poetry activities as emotionally calmer than conventional oral participation tasks:

*“When we discuss poems or read something emotional, it feels different from answering textbook questions. I feel more relaxed because there is not only one correct answer. You can explain your feeling or your opinion.”* (ST9, age 16)

Students repeatedly contrasted these experiences with the pressure associated with examination-focused instruction. One learner explained:

*“Usually English classes feel connected to marks all the time. You think about grammar, vocabulary, and whether your answer is correct. In drama activities, I feel like I can just communicate without worrying every second.”* (ST14, age 18)

Teacher accounts strongly supported these observations. Teachers described noticeable changes in classroom atmosphere during poetry- and drama-based lessons, particularly among students who were typically hesitant to participate orally. One teacher noted:

*“Students who are normally very quiet become more comfortable during drama activities. The classroom atmosphere changes because students stop focusing only on avoiding mistakes. They become more willing to try.”* (T2)

Another teacher emphasized the emotional shift that occurred when assessment pressure became less central:

*“In exam-oriented classes, students often speak with fear because they expect correction immediately. But when we use poetry or role-play, students seem more emotionally open. They participate more naturally because the activity feels less judgmental.”* (T5)

At the same time, emotional safety was not experienced uniformly by all learners. A smaller number of students reported that performance-based activities occasionally created different forms of anxiety, particularly when acting in front of peers. One participant commented:

*“Sometimes drama is enjoyable, but sometimes I still feel nervous because everyone is watching you. If you are shy, acting can feel uncomfortable at first.”* (ST7, age 17)

These contrasting experiences suggest that poetry and drama did not simply eliminate anxiety, but rather reshaped the emotional conditions under which classroom participation occurred. Students often described feeling less afraid of linguistic imperfection because creative activities shifted attention away from evaluation and toward interaction, interpretation, and shared participation.

#### 4.1.2 Enjoyment, relaxation, and emotional release

In addition to reduced fear of evaluation, many students described poetry and drama activities as emotionally enjoyable and mentally refreshing compared to routine classroom instruction. Learners frequently associated these activities with feelings of relaxation, freedom, and emotional release, particularly within the broader context of exam pressure and academic stress.

One student explained:

*“Most of our classes are serious because we are preparing for exams all the time. When we do drama or poetry, the classroom atmosphere changes. We laugh more, interact more, and I feel less mentally tired.”* (ST18, age 17)

Another participant described poetry discussions as emotionally meaningful because they allowed students to connect English learning with personal feelings and experiences:

*“Sometimes poetry lessons feel more human. We are not only studying language. We talk about emotions, situations, and experiences that feel real. That makes English feel less mechanical.”* (ST21, age 18)

Teachers similarly emphasized that poetry and drama often generated forms of enjoyment and emotional involvement that differed from conventional exam-oriented lessons. One teacher observed:

*“Students become emotionally engaged in a different way during these activities. Even students who usually look tired or disconnected become more active when they feel personally involved in the topic or role.”* (T4)

Another teacher noted that enjoyment appeared closely connected to opportunities for interaction and creativity:

*“Students often enjoy these lessons because they can move, speak, imagine, and cooperate with classmates. The classroom becomes more dynamic compared to lessons focused only on exercises or test preparation.”* (T1)

Importantly, enjoyment was not described merely as entertainment or temporary fun. Rather, participants frequently connected enjoyment to feelings of comfort, participation, and meaningful involvement. Several students suggested that poetry and drama created temporary relief from the repetitive and highly structured nature of exam-oriented learning.

These findings suggest that poetry- and drama-based instruction contributed to emotionally supportive classroom experiences by reducing fear of evaluation and fostering enjoyment, relaxation, and expressive participation. From a positive psychology perspective, such experiences appear to support foreign language enjoyment not simply through pleasure, but through emotionally meaningful and socially supportive forms of classroom engagement. At the same time, the findings also indicate that emotional safety remained contextually negotiated and shaped by individual learner differences, particularly in relation to shyness and performance-related discomfort.

### 4.2 Confidence, self-expression, and learner voice

#### 4.2.1 Speaking through roles and reduced self-consciousness

A second major theme concerned learners’ perceptions of increased speaking confidence during poetry- and drama-based activities. Many students described feeling more willing to speak English when classroom interaction became less centered on grammatical accuracy and more focused on communication, creativity, and participation. In particular, drama activities appeared to reduce self-consciousness by allowing learners to speak through fictional characters or imagined situations rather than directly as themselves.

One student explained:

*“When I have a role, I feel less embarrassed speaking English because it is not exactly me speaking. I am acting as someone else, so I stop thinking too much about how I sound.”* (ST6, age 17)

Another participant similarly described role-play as reducing the emotional pressure associated with oral participation:

*“In regular speaking activities, I become nervous because I feel everybody is judging my English. But in drama activities, everybody is participating together, so it feels easier to continue speaking without stopping all the time.”* (ST11, age 16)

For several learners, this reduced self-consciousness translated into greater willingness to communicate in English, including among students who typically avoided oral participation. One student reflected:

*“Usually I avoid speaking because I am afraid I will make mistakes or forget words. But during drama, I speak more because I am focused on the situation and not only on grammar. Sometimes I even surprise myself.”* (ST15, age 18)

Teachers also observed noticeable shifts in learners’ confidence during poetry- and drama-based activities. One teacher commented:

*“Students who are normally silent during textbook discussions often participate more actively during drama tasks. The role gives them some emotional protection, and this seems to reduce their fear.”* (T5)

Another teacher described confidence as emerging gradually through repeated participation:

*“Some students begin very hesitantly, but after several activities they become more comfortable speaking in front of others. They realize communication is possible even without perfect English.”* (T3)

At the same time, confidence development was not experienced equally by all students. A few participants indicated that performance-based activities could initially intensify feelings of vulnerability, particularly for learners who were already highly self-conscious. One student explained:

*“I became more confident later, but at the beginning I was uncomfortable because acting in front of classmates felt strange for me. It took time before I could relax.”* (ST2, age 16)

These accounts suggest that confidence was constructed relationally through participation, interaction, and emotional support rather than through linguistic mastery alone. Learners often described gaining confidence not because they believed their English had suddenly become perfect, but because the classroom environment temporarily reduced the emotional consequences of making mistakes.

#### 4.2.2 English as personal expression and identity exploration

Beyond speaking confidence, many students described poetry and drama as creating opportunities for self-expression and personal voice that differed substantially from conventional EFL instruction. Learners frequently contrasted creative activities with textbook-centered lessons, explaining that poetry and drama allowed them to communicate feelings, opinions, and experiences in ways that felt personally meaningful.

One participant reflected:

*“Sometimes in normal lessons, English feels only academic. You answer questions, memorize vocabulary, and prepare for exams. But with poetry or drama, I feel like I can express something about myself.”* (ST4, age 17)

Another student described poetry discussions as enabling emotional expression that was often absent from routine classroom activities:

*“When we talk about poems, people give different interpretations and emotions. It feels more personal. You are not only trying to find the correct answer from the textbook.”* (ST20, age 18)

Several learners also associated creative activities with a greater sense of ownership over English language use. One student explained:

*“In drama or poetry activities, I feel like English becomes more natural for me. I can use it to explain ideas, emotions, or experiences, not only school topics.”* (ST1, age 16)

Another participant articulated this shift in particularly reflective terms:

*“When I act or read poetry, I feel like English is not just a subject anymore. I feel I can communicate something real, not only repeat correct answers. Even if my grammar is imperfect, I still feel confident expressing myself.”* (ST4, age 17)

Teacher perspectives similarly emphasized the expressive dimension of poetry- and drama-based learning. One teacher observed:

*“Creative activities often reveal parts of students’ personalities that do not appear during normal lessons. Some students become much more expressive when they are given opportunities to speak creatively rather than academically.”* (T6)

Another teacher suggested that poetry and drama allowed students to engage with English in more personally meaningful ways:

*“Students sometimes begin to see English differently during these activities. Instead of viewing it only as an exam subject, they start using it to communicate feelings, opinions, and personal experiences.”* (T1)

At the same time, not all learners fully embraced opportunities for expressive participation. Some students preferred more structured classroom activities because creative tasks occasionally felt unfamiliar or emotionally exposing. One participant commented:

*“Sometimes I prefer clear exercises because with poetry or acting there is uncertainty. You have to express ideas in your own way, and that can feel difficult.”* (ST8, age 17)

These findings suggest that poetry and drama created classroom spaces in which English was experienced not only as an academic subject, but also as a medium for personal expression, interaction, and identity exploration. Learners frequently described feeling less constrained by accuracy-focused expectations and more able to experiment with voice, meaning, and participation. However, these experiences remained shaped by individual comfort levels and learners’ varying relationships with creative and expressive classroom practices.

### 4.3 Engagement within institutional and personal constraints

#### 4.3.1 Meaningful participation and sustained attention

A third major theme concerned learners’ experiences of engagement during poetry- and drama-based instruction. Many students described these activities as more stimulating, interactive, and mentally involving than routine exam-oriented lessons. Participants frequently explained that creative activities encouraged them to pay closer attention, participate more actively, and become more emotionally invested in classroom interaction.

One student reflected:

*“During normal lessons, sometimes I lose concentration because we repeat similar exercises again and again. But with drama activities, I stay focused because something is always happening and everybody is involved.”* (ST10, age 16)

Another learner described engagement as connected to interaction and unpredictability:

*“You do not know exactly what other students will say or how the scene will continue, so you pay attention more carefully. It feels more active than only listening to explanations.”* (ST13, age 17)

Several students also associated poetry and drama with improved memory and deeper involvement in language use. One participant explained:

*“I remember vocabulary and expressions more when they are connected to emotions or situations. If we act something or discuss a poem, the words stay in my mind longer.”* (ST19, age 18)

Another student emphasized the collaborative nature of engagement during creative activities:

*“In these lessons, everybody depends on each other more. You have to listen, respond, and continue the activity together. It feels less passive compared to regular classes.”* (ST5, age 17)

Teacher perspectives closely mirrored these observations. Teachers frequently described poetry- and drama-based activities as producing higher levels of participation and attentiveness than traditional textbook-centered instruction. One teacher noted:

*“Students often become more mentally present during these activities. Even students who are usually distracted participate more actively because the lesson feels more dynamic.”* (T1)

Another teacher linked engagement to opportunities for interaction and emotional involvement:

*“Poetry and drama encourage students to listen carefully to each other and react naturally. The classroom becomes more interactive, and students seem more emotionally connected to the lesson.”* (T6)

Importantly, engagement was often described as extending beyond simple enjoyment or entertainment. Participants frequently connected engagement with feelings of meaningful participation, cooperation, and personal involvement. Several students suggested that creative activities allowed them to experience English more actively rather than simply receiving information passively for examination purposes.

#### 4.3.2 Resistance, discomfort, and exam-oriented pressures

Although many participants described poetry and drama positively, the findings also revealed important tensions, limitations, and forms of resistance associated with creative classroom practices. Both students and teachers acknowledged that engagement with poetry and drama was shaped by individual learner preferences as well as broader institutional pressures connected to exam-oriented schooling.

Some students expressed discomfort with performance-based activities, particularly those involving acting in front of peers or improvisational speaking. One participant explained:

*“Sometimes I enjoy drama activities, but sometimes I become anxious because people are watching you closely. If you are naturally shy, it can still feel stressful.”* (ST7, age 17)

Another learner preferred more structured classroom activities because they felt predictable and academically safer:

*“I prefer lessons where I clearly know what is expected. With drama or poetry discussions, there is more uncertainty, and sometimes I worry about expressing myself incorrectly.”* (ST2, age 16)

A small number of students also questioned whether poetry and drama were always appropriate within highly exam-focused educational environments. One participant commented:

*“These activities are interesting, but sometimes students feel pressure because exams are very important. Some people think creative lessons take time away from preparation.”* (ST22, age 18)

Teachers similarly emphasized structural constraints that limited the regular integration of poetry and drama into upper secondary EFL classrooms. Several teachers described tensions between creative pedagogy and institutional expectations surrounding curriculum coverage and examination preparation. One teacher explained:

*“The biggest challenge is time. Teachers often feel pressure to complete the syllabus and prepare students for exams, so creative activities can become difficult to justify regularly.”* (T4)

Another teacher highlighted the institutional culture surrounding assessment:

*“In exam-oriented contexts, both students and parents often expect visible academic outcomes. Activities like poetry and drama may be viewed as less serious even when they support participation and confidence.”* (T2)

Teachers also noted that not all students responded positively to creative practices. One participant reflected:

*“Some students enjoy these activities immediately, while others resist because they are uncomfortable speaking publicly or expressing personal ideas. Teachers need to introduce these activities carefully and gradually.”* (T5)

These findings suggest that engagement with poetry- and drama-based instruction was shaped not only by the activities themselves, but also by broader classroom cultures, learner identities, and institutional realities. While many students experienced creative activities as emotionally supportive and engaging, these experiences remained negotiated within the constraints of examination pressure, performance anxiety, and differing learner preferences.

The findings indicate that poetry and drama functioned as psychologically meaningful classroom practices that created opportunities for enjoyment, confidence, self-expression, and participation. However, the findings also demonstrate that such practices operated within complex educational environments where emotional support, institutional expectations, and learner discomfort often coexisted simultaneously.

Although presented as distinct themes, the findings were closely interconnected across participants’ accounts. Experiences of emotional safety often appeared to support increased confidence and willingness to participate, while opportunities for self-expression contributed to deeper engagement with classroom activities. At the same time, these psychologically supportive experiences remained shaped by institutional expectations, examination pressures, and individual learner differences. Together, the findings suggest that poetry- and drama-based instruction functioned as emotionally and socially situated classroom practices through which adolescent learners negotiated participation, expression, and engagement in the foreign language classroom.

## 5 Discussion

This study explored the psychological dimensions of poetry- and drama-based instruction in adolescent EFL learning within an exam-oriented secondary education context in the United Arab Emirates. Drawing on students’ and teachers’ perspectives, the findings illustrate how creative language practices were experienced as emotionally meaningful, socially mediated, and institutionally situated classroom experiences. Across the three themes, poetry and drama were associated with emotional safety, increased confidence, opportunities for self-expression, and meaningful engagement. At the same time, these experiences remained shaped by performance anxiety, individual learner differences, and the broader realities of exam-oriented schooling. Interpreted through perspectives from positive psychology in SLA and sociocultural theory, the findings contribute a more contextually grounded understanding of how creative pedagogical practices may shape adolescent learners’ psychological relationship with the foreign language.

### 5.1 Emotional safety, enjoyment, and the social construction of foreign language experience

One of the clearest findings of the study was that many learners experienced poetry- and drama-based activities as emotionally safer and more enjoyable than conventional English lessons. Students repeatedly contrasted creative activities with highly evaluative classroom routines centered on correctness, testing, and fear of mistakes. These findings align with research in positive psychology in SLA, particularly work on foreign language enjoyment, which emphasizes the importance of emotionally supportive classroom experiences in sustaining participation and willingness to communicate (Dewaele and MacIntyre, 2014; Dewaele et al., 2019). However, the present findings extend previous research by showing that enjoyment was not experienced merely as entertainment or temporary classroom pleasure. Rather, enjoyment emerged through reduced fear of evaluation, collaborative participation, and opportunities for expressive interaction. Students often described feeling emotionally “lighter,” less monitored, and less constrained by accuracy-focused expectations during poetry and drama activities. In this sense, enjoyment appeared closely connected to emotional safety and perceived freedom from constant linguistic judgment. This finding is particularly significant in the context of upper secondary education, where learners are often navigating heightened academic pressure and increased sensitivity to peer evaluation (Steinberg, 2014). Within exam-oriented classrooms, language learning can become strongly associated with performance, correction, and measurable outcomes. The findings suggest that poetry and drama may temporarily disrupt these dynamics by creating classroom spaces in which experimentation, imagination, and participation become more legitimate forms of language use. Rather than removing difficulty from language learning, these activities appeared to reduce the emotional consequences associated with making mistakes.

The findings also contribute to sociocultural understandings of emotion in language learning. From this perspective, emotions are not simply internal psychological states, but are socially constructed through interaction, classroom norms, and participation in shared activities (Mercer and Dörnyei, 2020). Emotional safety in the present study emerged relationally through collaborative engagement, role-taking, and shared participation. Drama activities were especially important in this regard because speaking through fictional characters appeared to create emotional distance from direct personal evaluation. Consistent with Even (2011), learners frequently described feeling more comfortable taking linguistic risks when they were performing through a role rather than speaking directly as themselves.

Importantly, the findings also complicate overly positive interpretations of creative pedagogy. Although many learners experienced reduced anxiety and increased enjoyment, some students still described feelings of nervousness and discomfort, particularly during public performance activities. Emotional safety was therefore not universal or automatically produced through creative activities themselves. Instead, it remained negotiated through individual learner dispositions, classroom relationships, and perceptions of peer judgment. This nuance contributes to recent critiques within positive psychology in SLA that caution against treating positive emotional experiences as universally accessible or context-free (MacIntyre et al., 2019).

Overall, the findings suggest that poetry and drama may function as emotionally supportive pedagogical spaces within exam-oriented EFL classrooms. Their psychological value appears to lie not simply in making lessons more enjoyable, but in reshaping the emotional conditions under which learners participate in foreign language use.

### 5.2 Confidence, self-expression, and the development of learner voice

A second important finding concerns learners’ perceptions of increased confidence and expanded opportunities for self-expression during poetry- and drama-based activities. Students repeatedly described feeling more willing to speak, less constrained by grammatical accuracy, and more able to communicate ideas and emotions in personally meaningful ways. These experiences resonate strongly with research on self-efficacy and willingness to communicate in SLA, which suggests that learners’ perceptions of communicative capability strongly influence participation and engagement (Bandura, 1997; Gregersen and MacIntyre, 2014). At the same time, the findings extend existing understandings of confidence by illustrating its social and interactional nature. Learners did not describe confidence as emerging primarily from improved linguistic mastery or grammatical competence. Instead, confidence developed through participation in supportive communicative experiences where meaning, interaction, and creative expression were prioritized over accuracy alone. Many students explained that they spoke more freely because the classroom environment temporarily reduced the fear associated with linguistic imperfection. This supports sociocultural views of confidence as relationally constructed through interactional experience rather than individually possessed (Mercer and Dörnyei, 2020).

Drama activities appeared particularly important in reducing self-consciousness. Speaking through fictional roles enabled learners to distance themselves from direct personal exposure, which in turn encouraged greater willingness to communicate. This finding reinforces previous work suggesting that dramatic role-taking may create psychologically safer conditions for oral participation, especially among learners who experience anxiety during conventional speaking tasks (Even, 2011; Zhang, 2024). Importantly, however, learners’ accounts suggest that the value of role-play extended beyond reduced anxiety alone. Several students described drama as enabling them to experiment with different forms of expression, interaction, and participation that felt unavailable within more tightly structured classroom routines.

The findings also highlight the importance of voice and identity during adolescence. Learners frequently described poetry and drama as allowing English to become more personal, expressive, and emotionally meaningful. Rather than using English solely to demonstrate academic knowledge, students described using the language to communicate opinions, emotions, and experiences connected to their own lives. This aligns with Ushioda’s (2011) argument that language learning is closely connected to learners’ developing sense of self and agency.

The present findings contribute to this literature by showing how creative classroom practices may create temporary spaces for identity exploration within otherwise highly regulated educational environments. Poetry and drama appeared to legitimize forms of language use associated with imagination, interpretation, and subjective meaning rather than solely correctness and examination performance. In this sense, learners were not only practicing English, but also negotiating what it meant to participate as English users. At the same time, the findings suggest that opportunities for expressive participation were not equally comfortable for all learners. Some students preferred more structured and predictable classroom activities because creative expression occasionally produced uncertainty or emotional exposure. This highlights an important tension within expressive pedagogies. While opportunities for personal voice may empower some learners, they may simultaneously create discomfort for others, particularly within classroom cultures where students are accustomed to clearly defined academic expectations.

The findings suggest that poetry and drama may support adolescent learners’ confidence and learner voice not simply by increasing participation, but by reshaping the meaning of participation itself. Within creative activities, speaking English became associated less with demonstrating correctness and more with communicating meaning, emotion, and personal perspective.

### 5.3 Engagement, institutional tensions, and the limits of creative pedagogy

The third theme highlights the complex relationship between engagement, institutional expectations, and classroom realities in poetry and drama-based instruction. Many students and teachers described creative activities as more interactive, dynamic, and mentally involving than routine exam-oriented lessons. Learners frequently associated engagement with collaboration, unpredictability, emotional involvement, and opportunities for active participation. These findings support sociocultural perspectives that conceptualize engagement as emerging through participation in meaningful social activity rather than as a fixed learner trait (Vygotsky, 1978).

Importantly, the findings suggest that engagement in creative activities was connected not only to enjoyment, but also to perceived meaningfulness. Students often described feeling more mentally present because activities required interpretation, interaction, listening, and spontaneous response. In contrast to highly structured classroom routines focused on memorization and examination preparation, poetry and drama appeared to create spaces for more active and dialogic participation. This finding reinforces previous research suggesting that creative pedagogies may increase learner involvement by positioning students as active contributors to classroom meaning-making rather than passive recipients of information (Hanauer, 2012; Maley and Duff, 2005).

However, one of the most significant contributions of the present study lies in its attention to institutional tensions surrounding creative pedagogy. While previous studies have often emphasized the benefits of poetry and drama, the present findings illustrate how these benefits are continuously negotiated within exam-oriented educational systems. Teachers repeatedly described curricular pressure, limited instructional time, and assessment expectations as barriers to sustained implementation of creative practices. Similarly, some students questioned whether such activities were compatible with examination priorities or academically efficient within high-stakes learning environments. This finding extends existing research by demonstrating that institutional pressures do not simply restrict instructional variety; they also shape learners’ psychological experiences of classroom participation. In highly exam-oriented settings, students may internalize assumptions about what constitutes “serious” or “productive” learning. As a result, creative activities can become psychologically ambivalent spaces that simultaneously support engagement while also generating uncertainty about academic legitimacy. This institutional dimension was especially visible in students’ accounts of tension between enjoyment and exam preparation.

The findings therefore complicate simplified claims that creative pedagogies are universally engaging or transformative. Engagement remained uneven and contextually mediated. Some learners embraced collaborative and expressive participation, while others experienced discomfort, uncertainty, or performance anxiety. These differences highlight the importance of recognizing learner diversity within creative pedagogical approaches rather than assuming uniform psychological responses.

In this respect, the study contributes a more nuanced perspective to positive psychology in SLA by situating enjoyment, confidence, and engagement within broader institutional realities. The findings suggest that emotionally supportive and expressive classroom experiences cannot be fully understood independently of examination systems, classroom norms, and educational expectations. Poetry and drama may create opportunities for meaningful participation, but these opportunities remain shaped by the structural realities of schooling.

Overall, the discussion suggests that poetry- and drama-based instruction should not be viewed as universally effective alternatives to conventional pedagogy. Rather, these practices may be more productively understood as contextually situated pedagogical spaces that can support emotional safety, learner voice, and meaningful engagement when implemented sensitively within the constraints of real educational environments.

## 6 Conclusion, implications, and limitations

This qualitative study explored the psychological dimensions of poetry- and drama-based instruction in adolescent EFL learning within exam-oriented secondary school contexts in the United Arab Emirates. Drawing on focus group interviews with adolescent learners and semi-structured interviews with EFL teachers, the study examined how creative language practices were experienced emotionally, socially, and psychologically within upper secondary classrooms. The findings demonstrated that poetry and drama were experienced not simply as instructional techniques, but as psychologically meaningful classroom practices that shaped learners’ emotional safety, confidence, self-expression, and engagement with English language use.

From a positive psychology perspective, the findings highlight the importance of emotionally supportive classroom experiences in adolescent language learning. Poetry and drama frequently created conditions in which learners felt less constrained by fear of evaluation and more willing to participate meaningfully in classroom interaction. Importantly, the findings suggest that enjoyment in language learning emerged not merely through entertainment, but through reduced fear of mistakes, collaborative participation, and opportunities for expressive communication. At the same time, the study demonstrated that confidence and learner voice were socially negotiated through participation in supportive and interactive classroom practices rather than simply through linguistic proficiency alone.

The study also contributes to sociocultural understandings of language learning by illustrating how learners’ emotional experiences and participation were shaped through interaction, classroom norms, and institutional conditions. One of the central contributions of the study lies in demonstrating that the psychological value of poetry and drama-based instruction cannot be fully understood independently of the broader realities of exam-oriented schooling. While creative activities often supported engagement and emotional comfort, these experiences remained shaped by curricular expectations, examination pressures, learner preferences, and performance-related anxieties. The findings therefore contribute a more contextually situated understanding of creative pedagogy within adolescent EFL education.

Several pedagogical implications emerge from the study. For EFL teachers, poetry and drama may be integrated as psychologically supportive classroom practices that complement rather than replace exam-oriented instruction. Creative activities may help foster emotionally safer participation, encourage communicative experimentation, and create opportunities for self-expression among adolescent learners. However, the findings also suggest the importance of flexible and sensitive implementation. Not all learners experienced creative activities in the same way, and some students reported discomfort with public performance or open-ended expressive tasks. Teachers may therefore benefit from gradually introducing creative practices while remaining attentive to learner comfort, classroom dynamics, and varying participation preferences.

For curriculum designers and educational policymakers, the findings highlight the potential value of recognizing creative language practices not only as tools for linguistic development, but also as practices that may support learners’ emotional engagement, confidence, and sense of voice in the foreign language classroom. In highly exam-oriented educational systems, opportunities for emotionally meaningful and participatory language use may hold particular psychological significance for adolescent learners.

Several limitations should be acknowledged. The study was conducted within a specific educational and cultural context involving two secondary schools in the UAE, which may limit the transferability of the findings to other educational settings. In addition, the study relied primarily on interview-based accounts and did not incorporate classroom observations, which may have provided additional insight into interactional dynamics and classroom practices. The cross-language nature of the study also represents an important limitation. Although the Arabic-language focus group data were carefully translated and cross-checked, some emotional nuance and contextual meaning may have shifted during the translation process. Furthermore, as with all interpretive qualitative research, the analysis was shaped by the researchers’ theoretical perspectives and interpretive engagement with the data.

Future research could build on the present study by examining poetry- and drama-based instruction across different cultural and institutional contexts, incorporating longitudinal designs to explore changes in learners’ psychological experiences over time, or integrating classroom observations alongside interview data. Further research may also benefit from exploring how creative pedagogical practices are negotiated within educational systems characterized by strong examination cultures and institutional accountability pressures.

## Data Availability

The datasets presented in this article are not readily available because confidentiality reasons. Requests to access the datasets should be directed to edebreli@ciu.edu.tr.

## Appendices

### Appendix A

#### Student focus group interview guide

Opening prompt (warm-up):

- Can you briefly describe the kinds of poetry or drama activities you have experienced in your English classes?

Core focus group questions

1. Emotional experiences

- How did you usually feel during poetry or drama activities compared to regular English lessons?
- Were there moments when these activities felt enjoyable or relaxing? Can you describe one?
- Did you ever feel uncomfortable or nervous during these activities? Why?

2. Confidence and speaking

- Do you think poetry or drama activities changed how confident you feel speaking English in class?
- Were you more or less afraid of making mistakes during these activities? Can you explain?
- Did acting as a character or responding creatively make speaking easier or harder for you?

3. Engagement and participation

- How engaged did you feel during poetry or drama lessons compared to other English lessons?
- Did these activities help you pay more attention or participate more actively? In what way?
- Were there times when you felt bored or disconnected during these activities?

4. Self-expression and voice

- Did poetry or drama allow you to express your thoughts or feelings in English in a different way?
- Did these activities help you feel that English could be more personal or meaningful?
- Can you give an example of something you were able to express through poetry or drama?

5. Challenges and preferences

- What did you find difficult or uncomfortable about poetry or drama activities?
- Do you think these activities are suitable for all students? Why or why not?
- Would you like to have more, fewer, or the same number of poetry/drama activities in English classes?

Closing question:

- Is there anything else you would like to share about your experience with poetry or drama in learning English?

## Appendix B

### Teacher semi-structured interview guide

Core interview questions

1. Instructional background

- Can you describe how you typically use poetry and/or drama in your EFL classes?
- What motivated you to include these activities in your teaching?

2. Emotional and psychological impact

- How do students generally respond emotionally to poetry and drama activities?
- Do you notice differences in students’ anxiety or comfort levels during these lessons?
- Are there particular students who seem to benefit more emotionally from these activities?

3. Confidence and participation

- In your experience, do poetry and drama activities influence students’ speaking confidence?
- Have you observed changes in students’ willingness to participate or speak English?
- How do quieter or less confident students typically respond?

4. Engagement and classroom dynamics

- How do poetry and drama activities affect student engagement and attention?
- Do these activities change classroom interaction or peer relationships?
- How do students respond compared to more exam-oriented lessons?

5. Constraints and challenges

- What challenges do you face when using poetry or drama in secondary EFL classrooms?
- How do curriculum requirements or exam pressures influence your use of these activities?
- Are there students who resist or dislike these approaches? How do you handle this?

6. Reflections and recommendations

- What do you see as the psychological value of poetry and drama for adolescent learners?
- Would you recommend these practices to other EFL teachers? Why or why not?
- What conditions do you think are necessary for poetry and drama to be effective?

Closing prompt:

- Is there anything else you would like to add about your experiences using poetry or drama in EFL teaching?
